# Effect of milk phospholipids on the growth and cryotolerance of lactic acid bacteria cultured and stored in acid whey-based media

**DOI:** 10.3168/jdsc.2020-0007

**Published:** 2020-10-29

**Authors:** Lin Zhang, Israel García-Cano, Rafael Jiménez-Flores

**Affiliations:** Department of Food Science and Technology, Parker Food Science & Technology Building, The Ohio State University, Columbus 43210

## Abstract

Increasing interest in foods beyond their nutritional value has promoted the development of various novel functional foods that could convey multiple health benefits to consumers. The application of lactic acid bacteria (LAB) and milk phospholipids (MPL) in combination has shown some amplifying effects on the health benefits provided by both ingredients. Freezing is commonly used in LAB preservation and storage. However, the freezing/thawing process damages cell membranes and leads to a loss in viability and functionality of LAB. This study aimed to investigate the influence of MPL on growth and cryotolerance of LAB using acid whey-based medium (AWM) supplemented with 0.5% MPL. Fourteen LAB strains were initially screened from 124 LAB using acid whey-based medium (AW). We then evaluated the cell viability and acidification ability, using the plate counting method and skim milk fermentation test, respectively, of the 14 strains cultured in AW and AWM before and after 3 cycles of freezing/thawing. The presence of 0.5% MPL in AWM significantly promoted the growth of LAB. Supplementing the culture and storage medium with 0.5% MPL significantly enhanced the resistance of selected LAB to freeze-thaw cycles in terms of cell viability and acidification ability. These results suggest that supplementing with 0.5% MPL might promote the growth of LAB and enhance the cryotolerance of LAB cultures in fermented dairy products. This finding leads to a better understanding of the synergistic effects contributed by the LAB–MPL combination and promotes the development of new LAB–MPL functional products.

## Highlights

• Acidification activity is retained by adding milk phospholipids to acid whey-based medium
• Retention of activity (as rate of lactic acid production) is relevant to cryoprotection studies
• Acid whey from cottage cheese production is a good medium for preserving frozen cultures

## Graphical Abstract

**Figure:**
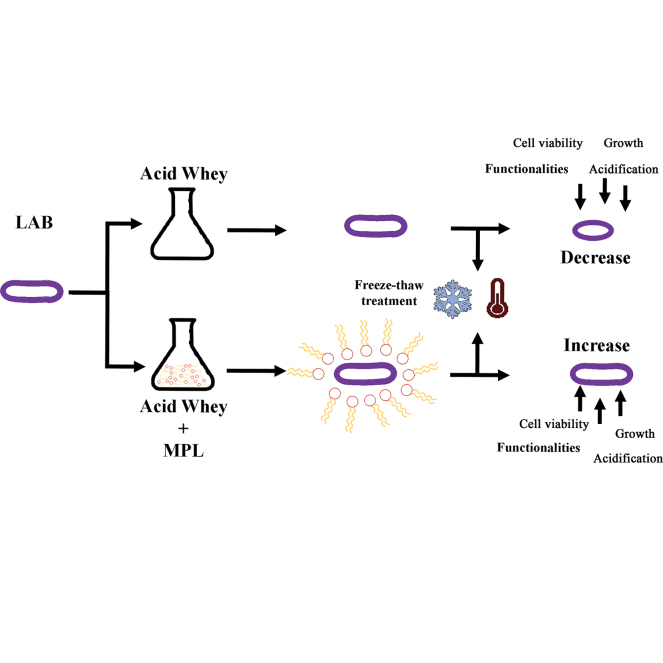
Summary: Supplementation of a lactic acid bacteria (LAB) culture medium with milk phospholipids (MPL) at low concentration (0.5%) results in a significant increase in viability and metabolic activity after freeze-thawing cycles. We speculate that the nature of the milk phospholipids maintains the integrity of the bacterial membranes and thus their metabolic function. This work demonstrates a feasible and economic way to enhance the cryotolerance of lactic acid bacteria, pointing toward possible means to develop functional foods and valorize acid whey.

Milk phospholipids (**MPL**) are valuable dairy components of the milk fat globule membrane and have been associated with various health benefits, including promoting neurological development, protecting against inflammation, and inhibiting cancer cell proliferation (Snow et al., 2011; Castro-Gómez et al., 2016; Bhinder et al., 2017). Recently, MPL became commercially available and have been used as functional ingredients (Arranz and Corredig, 2017).

Lactic acid bacteria (**LAB**) have a long history of being used as starter cultures and probiotics. The most used commercial media for LAB culture are M17 and de Man, Rogosa, and Sharpe (**MRS**) broth. However, these media are expensive and are used exclusively for laboratory research. Acid whey has the potential to be used as an inexpensive alternative medium for culture of microorganisms. Acid whey is a by-product of the manufacture of fresh, acid-coagulated cheese (e.g., cottage cheese, cream cheeses) or strained yogurt (e.g., Greek-style yogurt) (Manley, 2000; Alsaed et al., 2013), which would cause serious environmental problems if not used or disposed of properly (Dudkiewicz et al., 2016). Acid whey is a valuable dairy stream with functional proteins and peptides, lactose, vitamin, and minerals, and it has great potential to be used as the main component in microorganism culture media (Bylund, 2015).

Because of the health benefits and compatibilities of MPL and LAB with dairy products, using LAB and MPL in combination has gained the attention of researchers and shown some synergistic effects. The LAB–MPL combination can amplify the health benefits compared with using the ingredients separately. Morifuji et al. (2017) found that co-ingestion of LAB fermented milk and dietary sphingomyelin, a major group of MPL, increased the absorption of sphingomyelin in rats 2-fold, a significant increase compared with consuming sphingomyelin alone. Rocha-Mendoza et al. (2020) found that LAB previously cultured in MPL-supplemented medium exhibited greater adherence to Caco-2 cells. Moreover, the addition of 0.5 and 1% MPL into minimal medium promoted the growth of LAB, suggesting those LAB might utilize MPL as a source of energy or growth factors (Rocha-Mendoza et al., 2020). More investigations on the synergistic effects of the LAB–MPL combination are needed for the formulation of relevant functional products.

The optimal performance of LAB as both starter cultures and probiotics depends on the stabilization of their cell viability and metabolic activity. Frozen concentrates of LAB are extensively used in the manufacture of food, pharmaceutical, and dietary supplements. However, the freezing and thawing processes usually involved in preparation of frozen concentrates causes damage to cell membranes and leads to a loss in viability and functionality of LAB. The cryotolerance of LAB refers to the resistance of bacteria to the freeze-thawing process. Higher cryotolerance would drive better retention in cell viability and acidification ability of bacteria after freezing (Wang et al., 2005; Meneghel et al., 2017). Little is known about the effects of MPL on the growth and cryotolerance of LAB. Exogenous fatty acid (**FA**) sources have been shown to promote the growth of LAB and enhance their cryotolerance (Williams et al., 1947; Partanen et al., 2001; Tan et al., 2012). Because of the FA groups present in MPL and their amphiphilic nature, we hypothesized that addition of MPL into LAB-fermented dairy products would promote the growth and enhance cryotolerance of LAB. In the present study, we investigated the effects of MPL on the growth and cryotolerance of LAB cultured and stored in acid whey-based media as measured by cell viability and acidification ability after 3 cycles of freeze-thawing treatment. This study may extend the use of the LAB–MPL combination because MPL is a valuable dairy ingredient that may protect LAB in both starter cultures and probiotics.

Fresh acid whey obtained after cottage cheese making from the Superior Dairy Company (Canton, OH) was centrifuged at 10,000 × *g* for 20 min at 4°C (Sorvall Legend XT/XF Centrifuge; Thermo Scientific, Waltham, MA). After centrifugation, the supernatant was poured out and filtered using Whatman filter paper #1. The permeate was deproteinized by autoclaving at 121°C for 15 min, centrifuged, and filtered using the conditions as previously mentioned. Deproteinized whey was sterilized by autoclave at 121°C for 15 min to obtain basal acid whey. The prepared basal acid whey contained (wt/vol) 4.97 to 5.29% lactose, 0.55 to 0.69% protein, 0.06 to 0.08% fat, 0.48 to 0.52% ash, including 0.1 to 0.2% calcium. The prepared basal acid whey (**AW**) was supplemented with 0.5% (wt/vol) autoclaved yeast extract (Sigma-Aldrich, St. Louis, MO) and 0.5% (wt/vol) MPL (Fonterra, Auckland, New Zealand) to make the treatment medium, **AWM**. The MPL ingredient has a purity of 60%, so AWM contained 0.3 g of pure MPL per 100 mL of liquid medium. The MPL in AWM was replaced with the same amount of autoclaved reverse osmosis water (ThermoFisher Scientific, Waltham, MA) in the control medium (AW). The pH of the prepared AW and AWM was 4.5 to 4.6.

Normalized bacterial cultures of 124 LAB strains from the OSU-PECh culture collection (The Ohio State University, Columbus; García-Cano et al., 2019) were inoculated into AW by adjusting the initial absorbance (optical density) at 600 nm (**OD_600_**) to be 0.08 to 0.1. The bacteria cultures were added into a sterile 96-well plate and incubated inside the AccuSkan GO UV/Vis Microplate Spectrophotometer (Fisher Scientific, Pittsburgh, PA) at 37°C for 18 h with a 1-min pulsed medium shaking every 15 min. The maximum OD_600_ of each strain was recorded (assayed in duplicate). Fourteen representative LAB strains with a maximum OD_600_ >0.4 were selected for further experiments in this study: *Pediococcus acidilactici* OSU-PECh-1A; *Lactobacillus amylolyticus* OSU-PECh-23A; *Limosilactobacillus reuteri* OSU-PECh-33A, OSU-PECh-33B, OSU-PECh-35A, OSU-PECh-37A, OSU-PECh-48, OSU-PECh-50, OSU-PECh-81A, and OSU-PECh-92; *Streptococcus thermophilus* OSU-PECh-53; *Lacticaseibacillus paracasei* OSU-PECh-56; *Lacticaseibacillus rhamnosus* OSU-PECh-57A; and *Lactobacillus acidophilus* OSU-PECh-89.

Normalized bacteria cultures of those 14 selected LAB strains were inoculated into 5 mL of AW and AWM, respectively, with an initial OD_600_ of 0.08 to 0.1. The bacteria cultures were incubated at 37°C for 24 h. Then, bacteria were transferred one more time and inoculated 0.1% (vol/vol) into fresh AW or AWM and incubated at 37°C for 48 h. After incubation, viable counts were done using MRS (Difco/Becton Dickinson Co., Sparks, MD) agar plates. The plates were incubated at 37°C for 36 h before counting. All bacteria were assayed in duplicate, and all plates were inoculated in triplicate.

The freeze-thawing treatment was modified from Meneghel et al. (2017). One milliliter of bacterial culture obtained after culturing in AW or AWM was transferred into a 2-mL sterile cryogenic tube (Fisher Scientific). The tube was immediately frozen at −80°C for 23 h and then thawed at room temperature for 1 h before conducting the next freeze-thawing cycle and analysis. The freeze-thawing process was repeated 3 times before performing the following experiments.

As described by Meneghel et al. (2017), LAB counts were calculated before and after freeze-thawing cycles treatment. Counts (in cfu/mL) were conducted using the surface plating method. Samples were serially diluted in saline solution (0.85% NaCl, pH = 7.0) and appropriate dilutions, determined by preliminary experiments, were spread on MRS agar plates. All bacteria were assayed in duplicate; 2 dilutions and a triplicate for each dilution were tested for each sample. The plates were incubated at 37°C for 48 h before counting. Only plates with counts in the range of 30 to 300 cfu counted toward the final calculation. The retention in viability (%) was calculated as follows:

$$\text{Viability}=\frac{\text{log}{\left[\text{cfu}/\text{mL}\right]}_{\text{after treatment}}}{\text{log}{\left[\text{cfu}/\text{mL}\right]}_{\text{before treatment}}}\times 100\%,$$

where $\text{log}{\left[\text{cfu}/\text{mL}\right]}_{\text{before treatment}}$ refers to bacterial cell concentration obtained before the freeze/thaw cycle treatment, and $\text{log}{\left[\text{cfu}/\text{mL}\right]}_{\text{after treatment}}$ refers to cell concentration determined after the treatment.

The rate of pH decrease in skim milk (Difco/Becton Dickinson Co.) after addition of bacterial cultures was measured to evaluate the retention of acidification activity, according to Meneghel et al. (2017) with modifications. Twenty milliliters of skim milk (Difco/Becton Dickinson Co.) was reconstituted from dry powder at 100 g/L, sterilized by autoclaving, and warmed to 37°C in an incubator. The bacteria after 3 cycles of freeze-thaw were assayed by using 4 mL of the corresponding bacterial culture as inoculum in 20 mL of sterile skim milk medium, and the pH was measured directly after 4 h of fermentation at 37°C. Acidification activity (%) was calculated as follows:

$$\text{Acidification activity}=\frac{{\left[\text{pH}/\text{decrease}\right]}_{\text{after treatment}}}{{\left[\text{pH}/\text{decrease}\right]}_{\text{before treatment}}}\times 100\%,$$

where ${\left[\text{pH}/\text{decrease}\right]}_{\text{before treatment}}$ refers to the decrease in pH of the skim milk inoculated with the bacterial cultures before the freeze/thaw cycle treatment, and ${\left[\text{pH}/\text{decrease}\right]}_{\text{after treatment}}$ refers to the decrease in pH of the skim milk inoculated with bacterial cultures after treatment.

To compare bacterial growth in AW and AWM and viability and acidification ability after freeze-thawing treatment, we conducted a linear mixed-effect model with treatment as a fixed effect and bacteria as a random effect, with an *F*-test for each experiment (R software version 3.6.1 by lme4 package, https://www.r-project.org/). The criterion for significance of all tests was set at *P* < 0.05.

The growth of LAB cultured using AW varied widely, as shown in Figure 1. A similar acid whey-based medium prepared in another study was found to create favorable conditions for the growth of some strains but not all because of its incomplete nutrient profile and low pH (4.5–4.6 in this study; Mondragón-Parada et al., 2006; Dudkiewicz et al., 2016). Usually, dominant dairy products that deliver LAB, such as yogurt and other fermented milk, have a relatively low pH (Ranadheera et al., 2010). Our prepared AW was similar to conditions that LAB would encounter in fermented dairy products and hence was appropriate for screening of LAB in our study. Fourteen LAB strains with a final OD_600_ >0.4 were selected for later experiments. Because selected LAB could grow in prepared AW (with a final OD_600_ >0.4), we could observe the effect of supplemented MPL on those LAB. In contrast, if LAB with final OD_600_ <0.4 were selected, they would not grow well in prepared AW, and we could not observe the effects of MPL on those bacteria. These 14 strains were chosen because they were representative of growth in AW medium.

**Figure 1:**
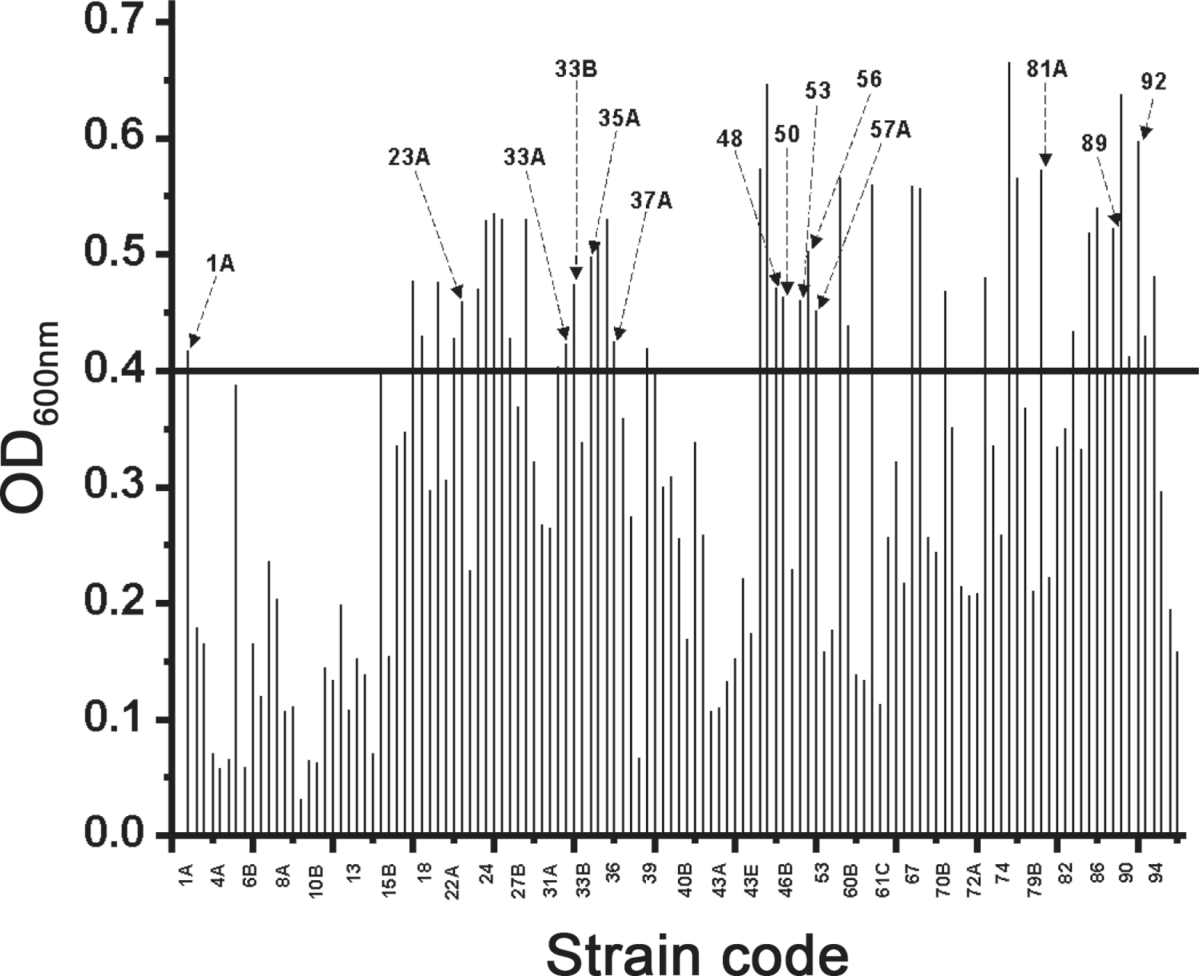
Growth of lactic acid bacteria in the acid whey-based medium. OD = optical density. Representative bacterial strains selected in this study are shown by arrows above the bars and identified as follows: 1A = *Pediococcus acidilactici* OSU-PECh-1A; 23A = *Lactobacillus amylolyticus* OSU-PECh-23A; 33A = *Limosilactobacillus reuteri* OSU-PECh-33A; 33B = *L. reuteri* OSU-PECh-33B; 35A = *L. reuteri* OSU-PECh-35A; 37A = *L. reuteri* OSU-PECh-37A; 48 = *L. reuteri* OSU-PECh-48; 50 = *L. reuteri* OSU-PECh-50; 53 = *Streptococcus thermophilus* OSU-PECh-53; 56 = *Lacticaseibacillus paracasei* OSU-PECh-56; 57A = *Lacticaseibacillus rhamnosus* OSU-PECh-57A; 81A = *L. reuteri* OSU-PECh-81A; 89 = *Lactobacillus acidophilus* OSU-PECh-89; 92 = *L. reuteri* OSU-PECh-92.

Statistical analysis of the bacterial count data showed that AWM resulted in a higher bacterial concentration than culture in AW (*P*-value = 0.002), indicating that the presence of 0.5% MPL in AWM significantly promoted growth of LAB. Previous studies have shown that a low concentration of medium- or long-chain UFA promoted growth of LAB, whereas a high concentration of long-chain SFA had inhibitory effects (Williams et al., 1947; Partanen et al., 2001). The AW medium had a limited amount of fat (0.06–0.08%). Milk phospholipids are defined as “lipids containing phosphorus,” with an amphiphilic structure consisting of a hydrophilic phosphorus head and a hydrophobic tail containing FA, mainly UFA (Contarini and Povolo, 2013; Ortega-Anaya and Jiménez-Flores, 2019). Hence, we might speculate that there are similarities between the effects of fats and FA on LAB and those caused by MPL, so it is reasonable that the supplementation of AW with 0.5% MPL promoted growth. Another study found that the addition of 1% milk fat into Cheddar cheese resulted in a higher final cell density of *Lacticaseibacillus casei*(Tan et al., 2012).

After 3 freeze-thaw cycles, the viability of most tested LAB (92%) previously cultured in both AW and AWM was >70% (Figure 2), indicating good retention of viability of those tested LAB isolated from fermented dairy products in general. Statistical analysis of viability data demonstrated that bacteria cultured and stored in AWM had significantly higher viability than the same strain cultured and stored in AW (*P* = 0.0123). Viability is a critical factor when incorporating probiotic LAB into dairy products. An adequate amount of viable probiotic cultures should be present in probiotic products throughout their shelf life to be able to confer health benefits to the host. It is generally accepted that for the probiotic effect to be transferred to the host, products should have a minimum concentration of 10^6^ cfu/mL or 10^6^ cfu/g, and that a total of 10^8^ to 10^9^ probiotic microorganisms should be consumed daily (Kechagia et al., 2013). However, retention in metabolic activities cannot be reflected by cell viability: a bacterium can remain viable but lose its functional activity.

**Figure 2:**
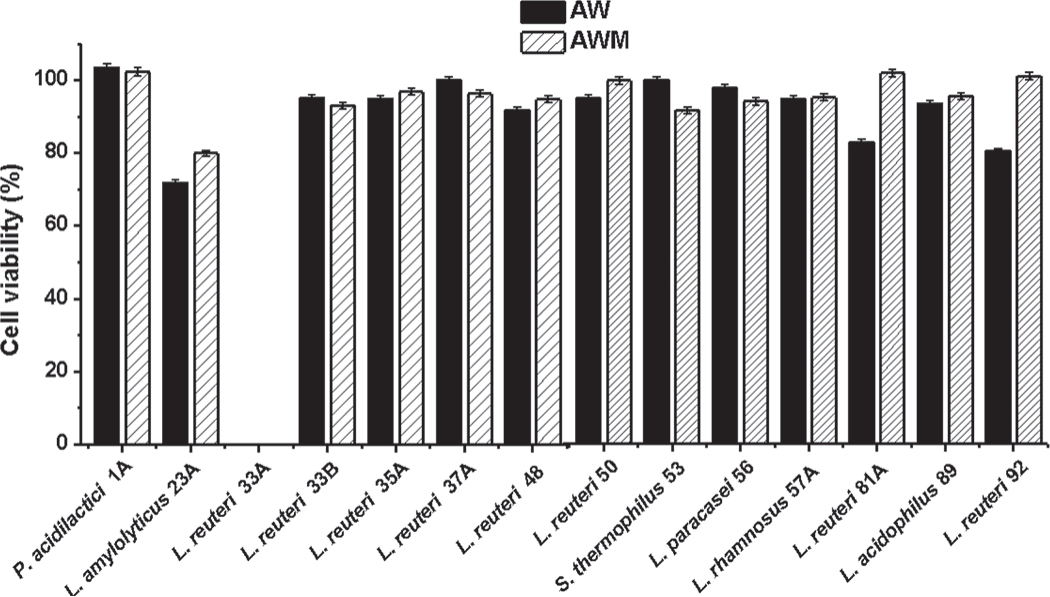
Comparison of cell viability of lactic acid bacteria (LAB) cultured and stored in acid whey-based medium (AW) and acid whey-based medium supplemented with 0.5% milk phospholipids (AWM) after freeze-thawing treatment. Strains: *Pediococcus acidilactici* OSU-PECh-1A, *Lactobacillus amylolyticus* OSU-PECh-23A, *Limosilactobacillus reuteri* OSU-PECh-33A, *L. reuteri* OSU-PECh-33B, *L. reuteri* OSU-PECh-35A, *L. reuteri* OSU-PECh-37A, *L. reuteri* OSU-PECh-48, *L. reuteri* OSU-PECh-50, *Streptococcus thermophilus* OSU-PECh-53, *Lacticaseibacillus paracasei* OSU-PECh-56, *Lacticaseibacillus rhamnosus* OSU-PECh-57A, *L. reuteri* OSU-PECh-81A, *Lactobacillus acidophilus* OSU-PECh-89, and *L. reuteri* OSU-PECh-92. Error bars are standard deviations.

The retention of acidification ability, one important metabolic activity of LAB, showed a wide range from 0 to 100% among tested strains (Figure 3). Statistical analysis of the acidification ability data demonstrated that bacteria cultured and stored in AWM retained acidification ability significantly better than the same strain cultured and stored in AW (*P* < 0.0001). The various degrees of damage to LAB caused by freeze-thaw cycles have been reported previously (Meneghel et al., 2017).

**Figure 3:**
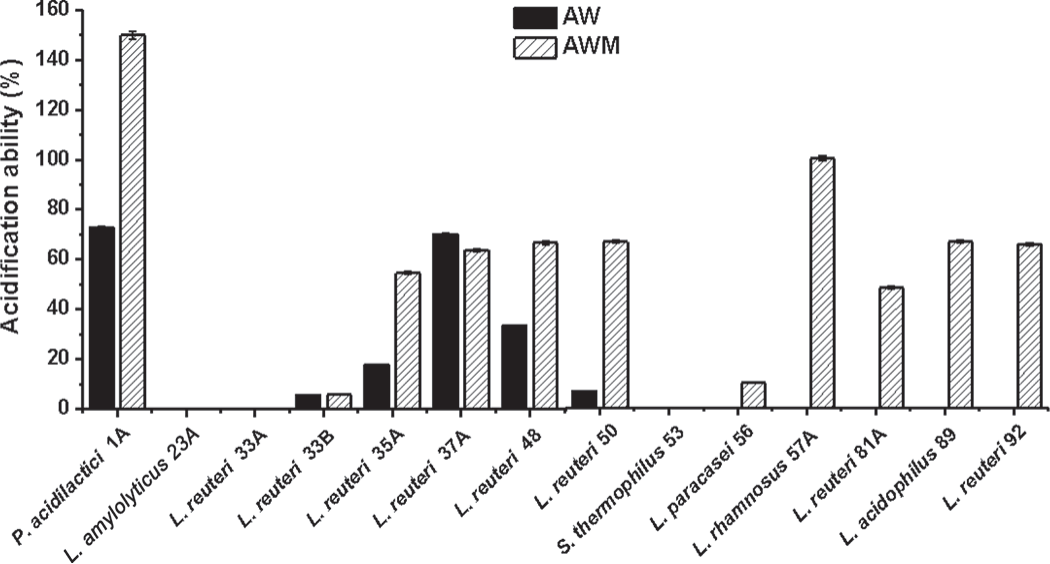
Comparison of acidification activity of lactic acid bacteria (LAB) cultured and stored in acid whey-based medium (AW) and acid whey-based medium supplemented with 0.5% milk phospholipids (AWM) after freeze-thawing treatment. Strains: *Pediococcus acidilactici* OSU-PECh-1A, *Lactobacillus amylolyticus* OSU-PECh-23A, *Limosilactobacillus reuteri* OSU-PECh-33A, *L. reuteri* OSU-PECh-33B, *L. reuteri* OSU-PECh-35A, *L. reuteri* OSU-PECh-37A, *L. reuteri* OSU-PECh-48, *L. reuteri* OSU-PECh-50, *Streptococcus thermophilus* OSU-PECh-53, *Lacticaseibacillus paracasei* OSU-PECh-56, *Lacticaseibacillus rhamnosus* OSU-PECh-57A, *L. reuteri* OSU-PECh-81A, *Lactobacillus acidophilus* OSU-PECh-89, and *L. reuteri* OSU-PECh-92. Error bars are standard deviations.

The effects of freeze-thaw cycles on LAB include inhibiting the growth of LAB, reducing or interrupting their metabolic activity, and even microorganism death, which affects the viability and functional properties of incorporated probiotic LAB (Alamprese et al., 2002). Modification of culture conditions; for example, temperature (Murga et al., 2000), pH (Wang et al., 2005), nutrients (Panoff et al., 2000), and the addition of cryoprotective agents to storage media or food products (e.g., glycerol, Fonseca et al., 2006; inulin, Rezaei et al., 2014), has been shown to improve the cryotolerance of LAB. Studies have also suggested that most LAB could incorporate exogenous FA such as oleic acid (Partanen et al., 2001; Tan et al., 2012) and PUFA (Kankaanpää et al., 2004) into their membranes, which could increase the fluidity of the membrane and thus protect the LAB against damage caused by freeze-thawing and extreme temperature (Liu and Hofmann, 1962; Smittle et al., 1974; Partanen et al., 2001). Membrane fluidity has been correlated with storage temperature and a combination of different FA structures, including different saturation levels and chain lengths (Meneghel et al., 2017). Higher membrane fluidity provides bacteria with greater resistance to freezing and thawing stress (Kwon et al., 2018). Tan et al. (2012) found that the addition of 1% milk fat into Cheddar cheese would change the cytoplasmic membrane FA composition of *Lacticaseibacillus casei*. Because MPL are polar fractions of milk fat, LAB might be able to incorporate certain FA from the supplemented MPL, resulting in a change in cell membrane FA composition, and thus acquire higher cryotolerance. The cell membrane FA composition of MPL-treated LAB would be an interesting topic for future research. We think that this effect due to the interaction with MPL is strain-specific in the case of LAB, and likely also has some specificity in other bacteria.

In this study, AW medium is a way of utilizing acid whey and could support the growth of some LAB with a final OD_600_ >0.4, which is high enough to observe the effect of MPL on LAB. Moreover, AW medium is similar to commercial fermented dairy products, so it is more appropriate for assessing effects of MPL than MRS medium. The results of this study support the conclusion that addition of 0.5% MPL to AW promotes the growth and enhances the cryotolerance of LAB significantly, specifically in terms of cell viability and acidification ability. This study may lead to a better understanding of the synergistic effects of using MPL and LAB in combination and may contribute to the production of novel functional products.
